# Supplementary material for: Integrative analysis of the shikonin metabolic network identifies new gene connections and reveals evolutionary insight into shikonin biosynthesis
Source: Hortic Res. 2022 Jan 20;9:uhab087. doi: 10.1093/hr/uhab087 (PMC8969065; doi:10.1093/hr/uhab087)
Supplement: Web_Material_uhab087 [file web_material_uhab087.zip › Supplemental Figures S1-S21.pdf]

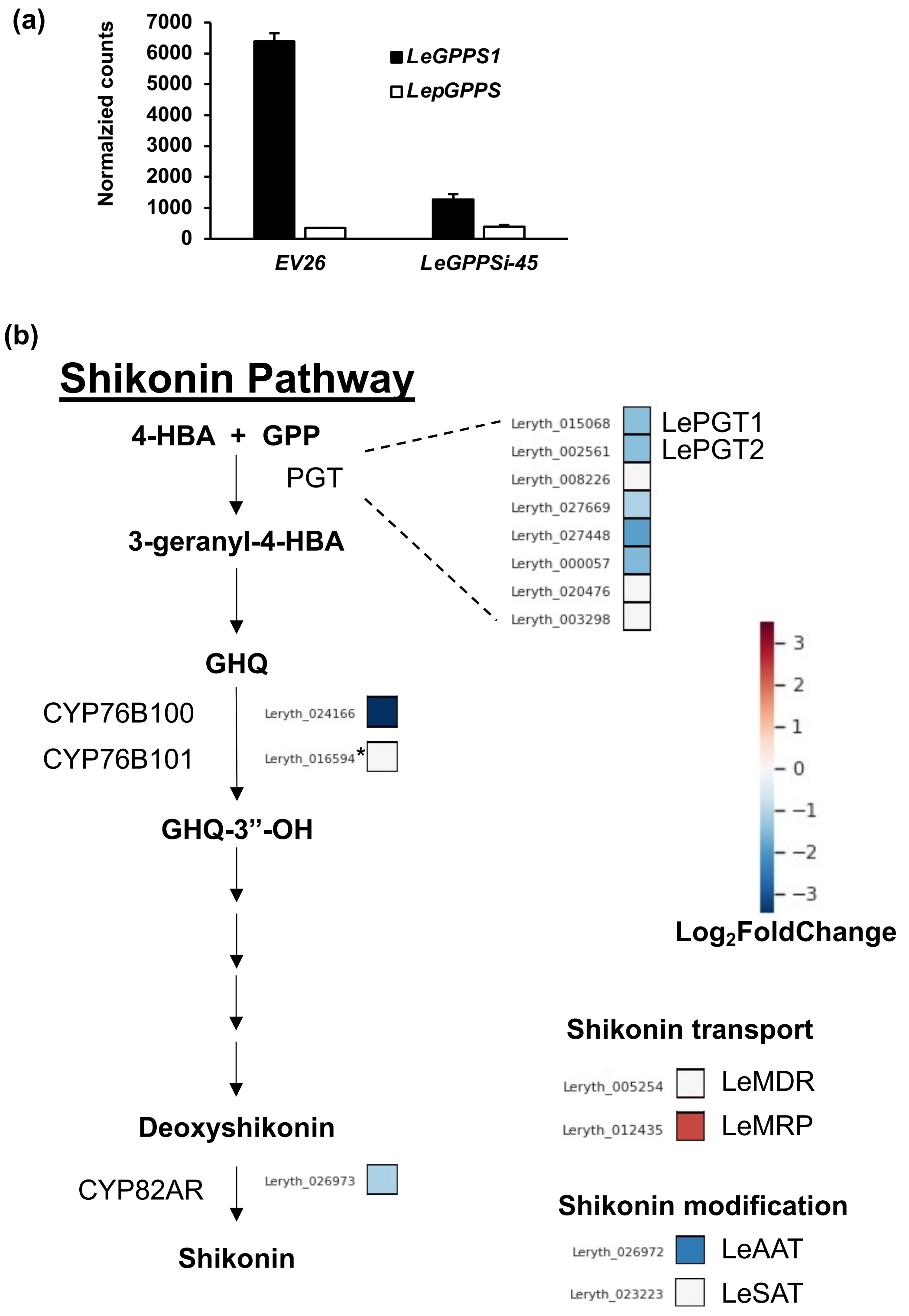

Fig. S1 Effect of *LeGPPS* RNAi downregulation on expression of shikonin pathway genes. The average $\log_2$fold-change in expression for each gene in *LeGPPSi*-45 lines compared to *EV-26* lines. Expression levels of *LeGPPS* and the canonical plastid-localized GPPS gene (*LepGPPS*) (a). Expression levels of shikonin pathway genes (b). The CYP76B101 gene (*) was not included in this DE analysis as the non stranded RNAseq library was unable to distinguish between CYP76B101 (Leryth_016594) and Leryth_016593, which occupies the same genomic location but is encoded on the opposite strand. See Fig. 1 legend for abbreviations and Table S4 for gene descriptions.

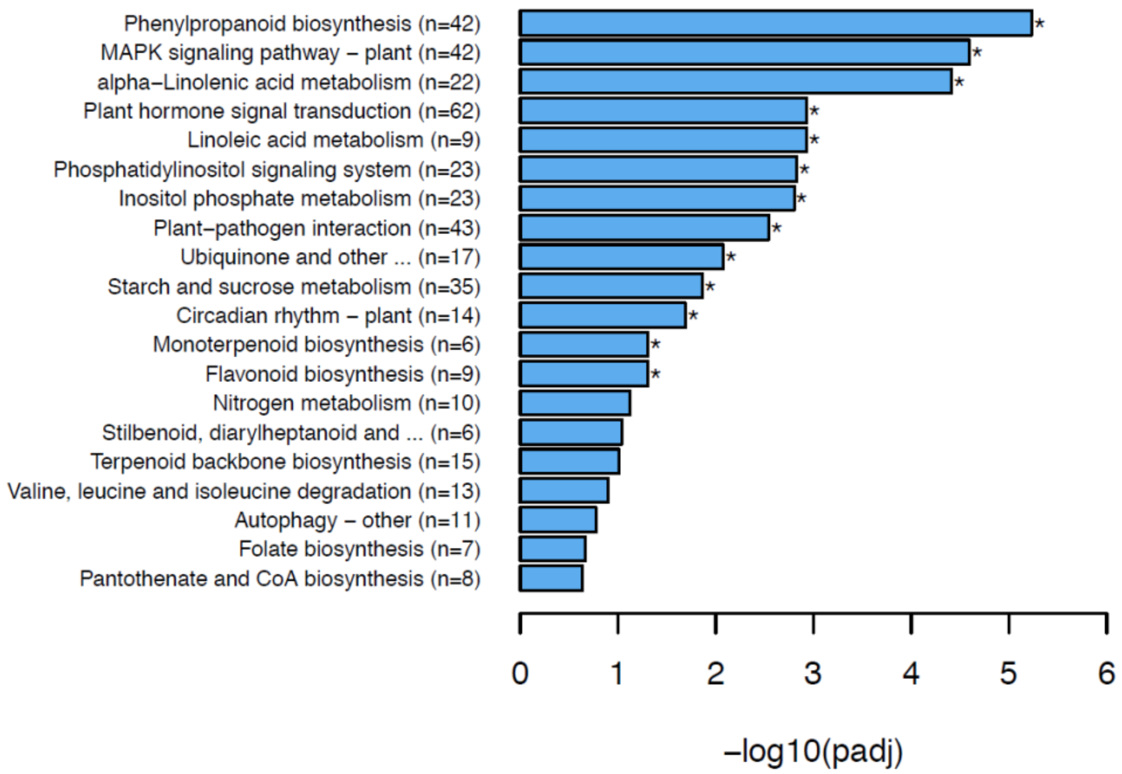

Fig. S2 Kyoto Encyclopedia of Genes and Genomes (KEGG) term enrichment analysis of genes downregulated in *LeGPPSi*-45 compared to *EV*-26 hairy root lines. KEGG pathways with corrected p-value < 0.05 were considered significantly enriched by differential expressed genes. *EV*-26, empty-vector control line 26; *LeGPPSi*-45, *LeGPPS*-RNAi line 45.

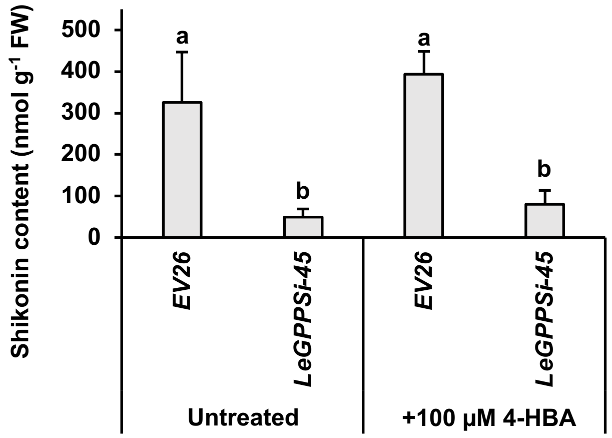

Fig. S3 Exogenous application of 4-hydroxybenzoate (4-HBA) does not restore shikonin production in *LeGPPSi*-45 lines. All data are means ± SEM (n = 4 biological replicates). Different letters indicate significant differences via analysis of variance (ANOVA) followed by post-hoc Tukey test (α= 0.05).

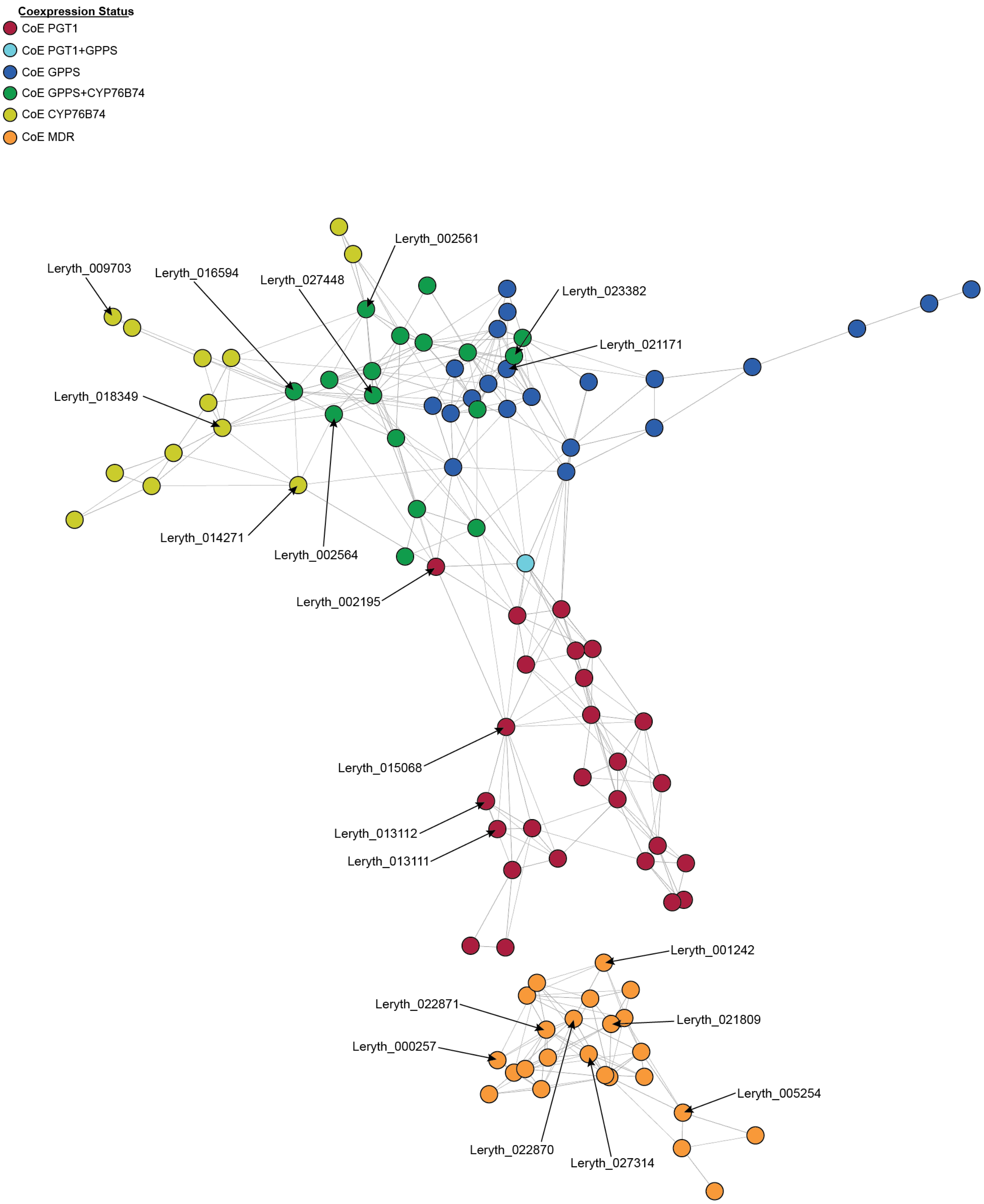

Fig. S4a Shikonin subnetwork N1. Network map of genes coexpressed with *LePGT*, *LeGPPS*, *LeCYP76B101*, and *LeMDR* using the N1 global coexpression network. Nodes are colored according to the gene's coexpression status with known shikonin genes. Network maps were drawn using a Fruchterman-Reingold force-directed layout using the edge-weighted spring embedded layout in cytoscape (https://cytoscape.org).

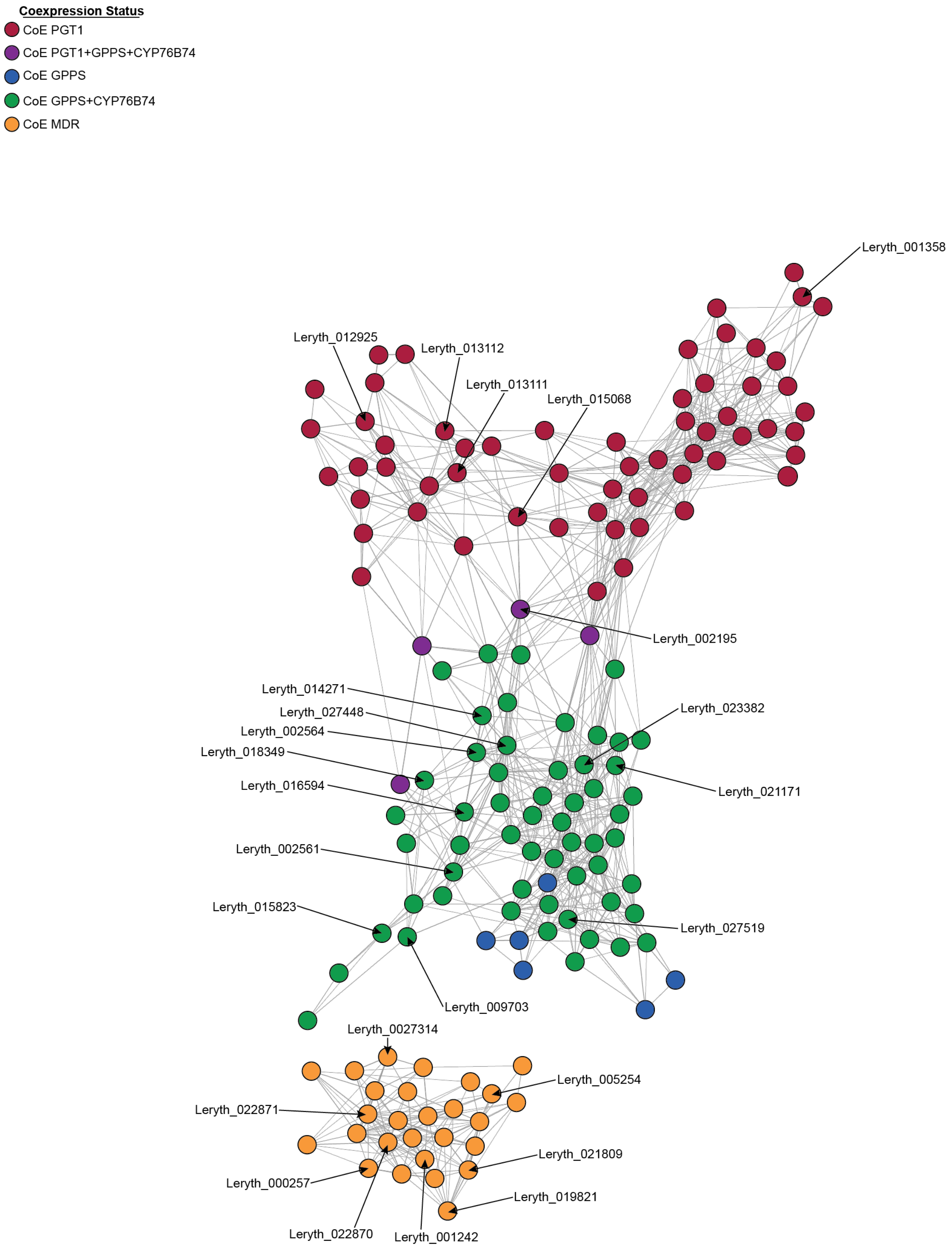

Fig. S4b Shikonin subnetwork N2. Network map of genes coexpressed with *LePGT*, *LeGPPS*, *LeCYP76B101*, and *LeMDR* using the N2 global coexpression network. Network maps are drawn as described in Supplemental Figure S1a.

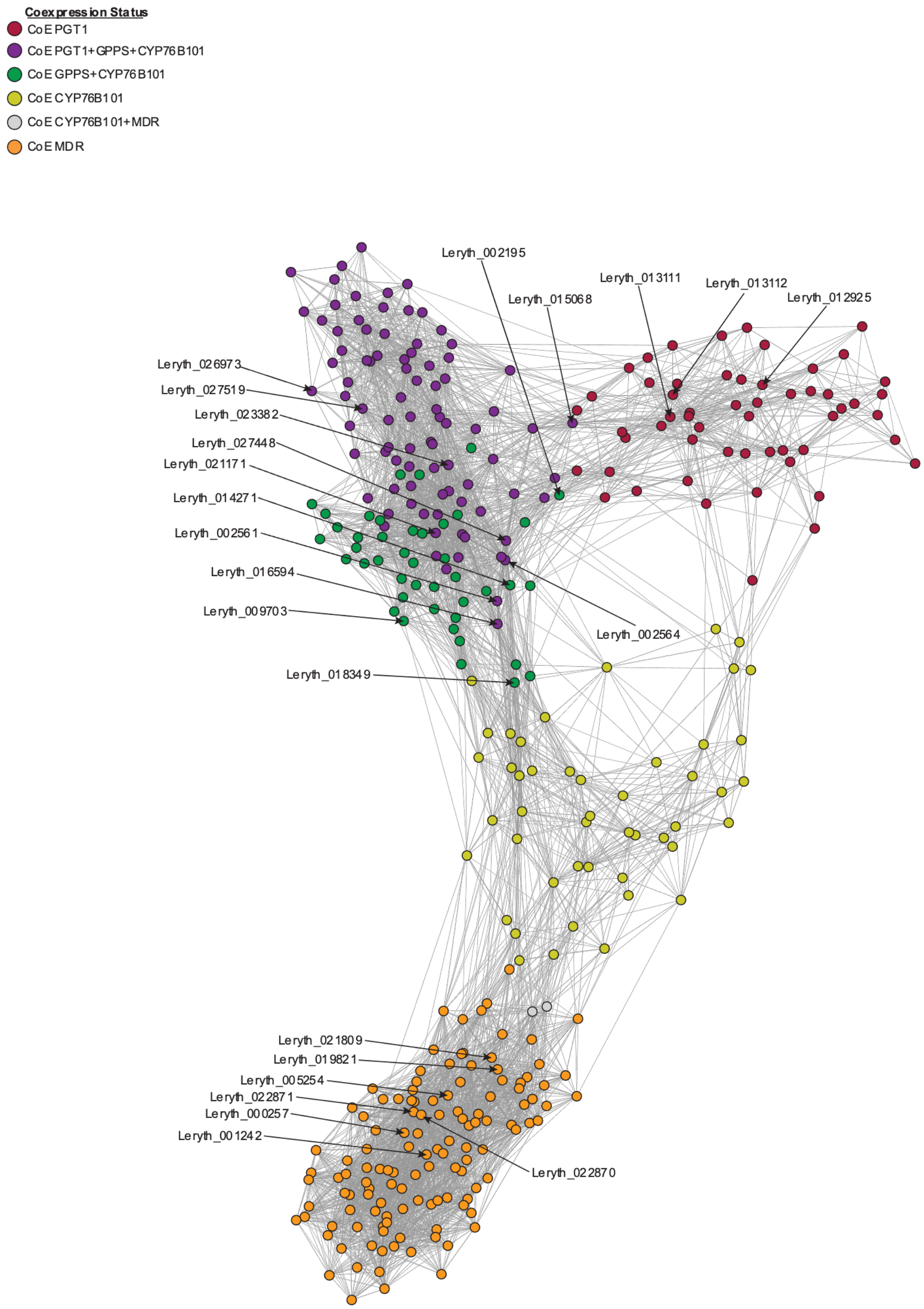

Fig. S4c Shikonin subnetwork N3. Network map of genes coexpressed with *LePGT*, *LeGPPS*, *LeCYP76B101*, and *LeMDR* using the N3 global coexpression network. Network maps are drawn as described in Supplemental Figure S1a.

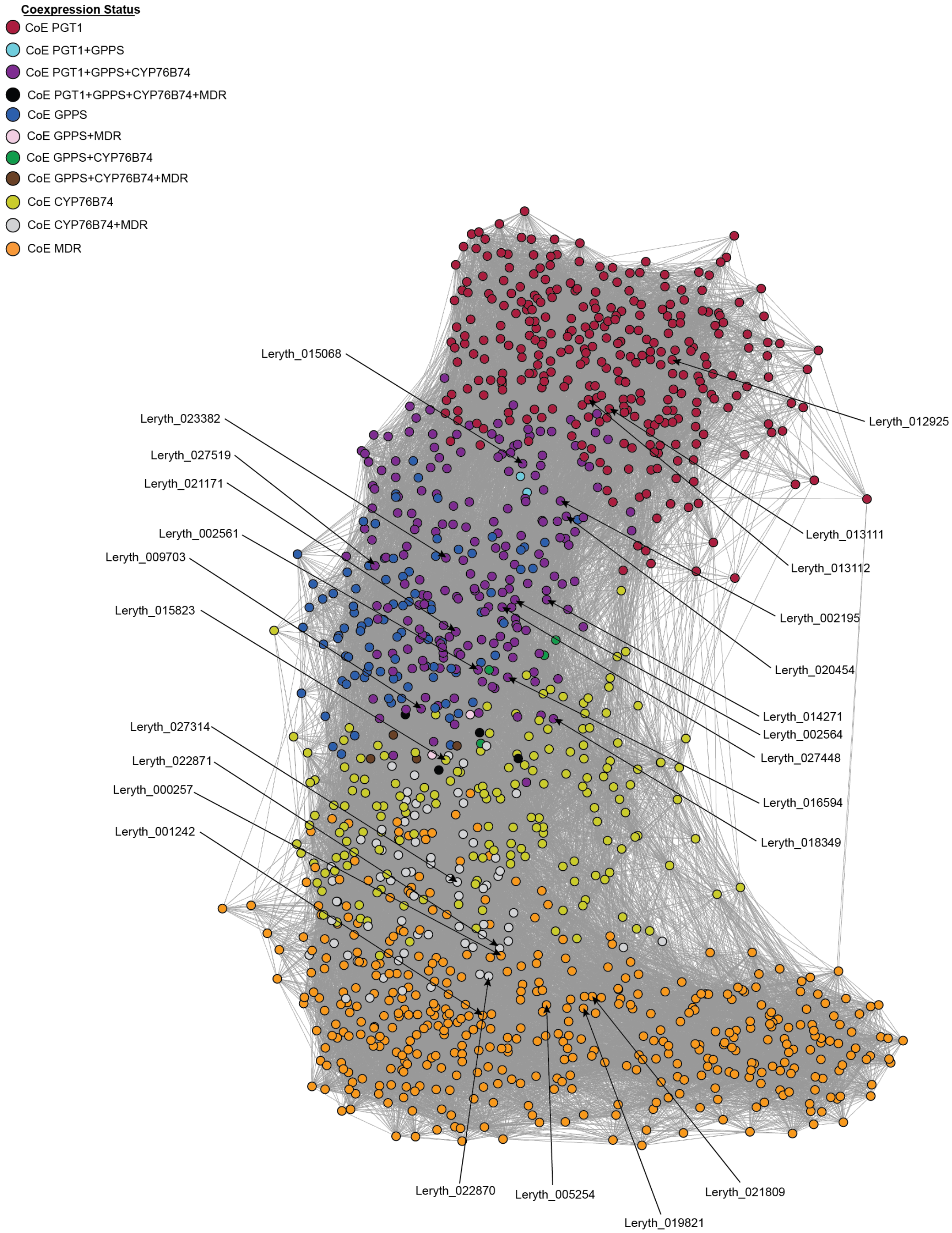

Fig. S4d Shikonin subnetwork N4. Network map of genes coexpressed with *LePGT*, *LeGPPS*, *LeCYP76B101*, and *LeMDR* using the N4 global coexpression network. Network maps are drawn as described in Supplemental Figure S1a.

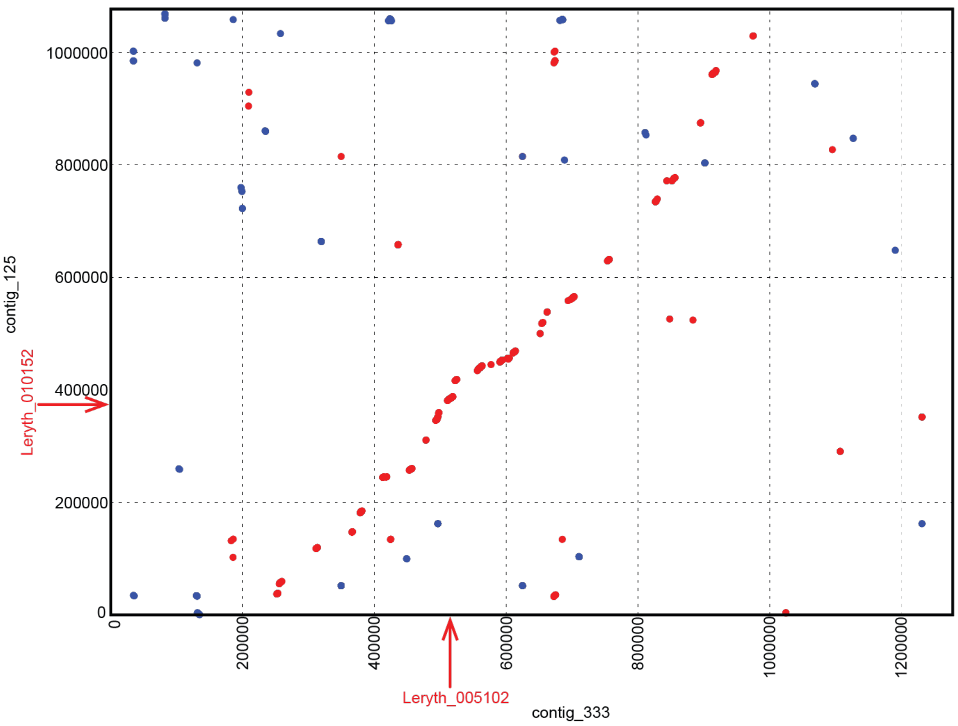

Fig. S5 Shared synteny between FPPS homologs. Mummerplot of syntenic block containing farnesyl pyrophosphate synthase homologs Leryth_005102 (*LeFPPS1*) and Leryth_010152 *(LeFPPS3*). The locations of the homologs are indicated by the red arrows.

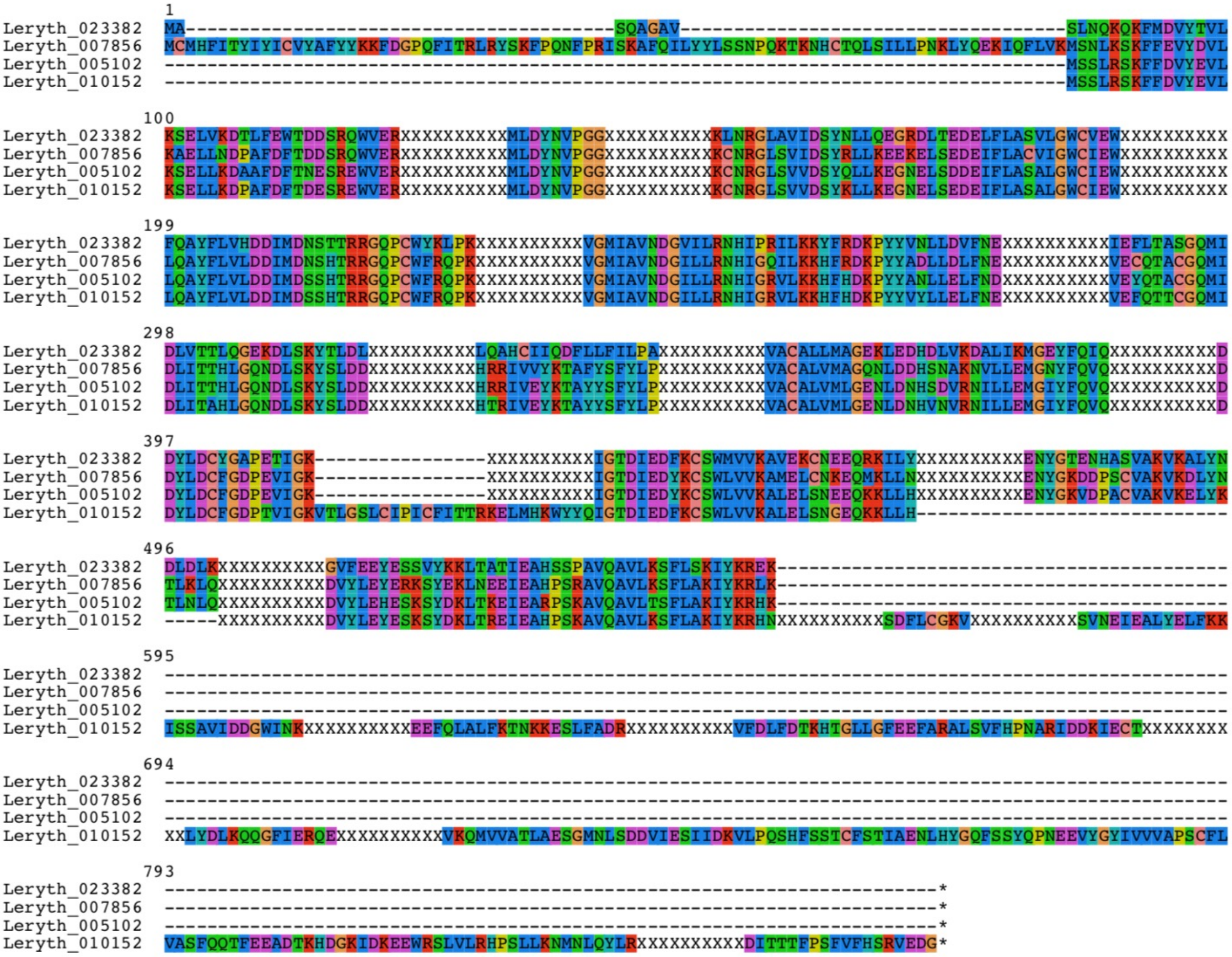

Fig. S6 Conservation of intron locations in the *FPPS* gene family. Multiple sequence alignment showing conservation of intron locations (indicated by xxxxxxxxxx) between LeGPPS (Leryth_023382), LeFPPS1 (Leryth_005102), LeFPPS2 (Leryth_007856), and LeFPPS3 (Leryth_010152).

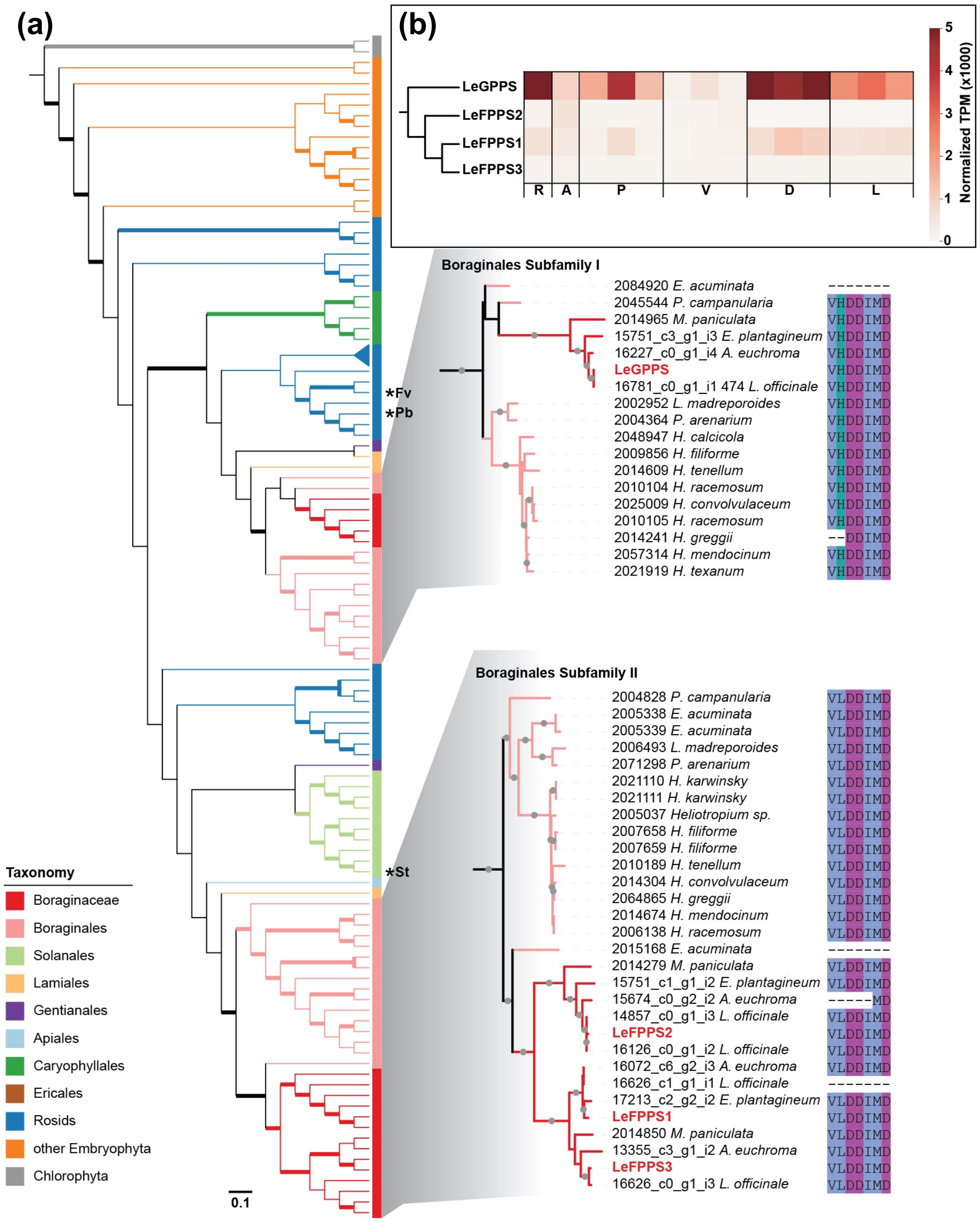

Fig. S7 Phylogenetic analysis and expression profile of FPPS genes. Maximum likelihood tree of FPPS gene family in green plants (a). In the full cladogram, left, nodes with IQ-TREE support values > 95 are bolded. The branches and outer color bar are color-coded to match the taxonomic classification of each sequence. The tree is rooted on Chlorophytes. Non-Boraginales sequences containing a Histidine residue adjacent to the conserved Asp-rich motif are indicated by an asterisk (Fv, *Fragaria vesca*; Pb, *Pyrus bretschneideri*; St, *Solanum tuberosum*). Detailed phylograms of the two Boraginales clades, right, along with an alignment of Asp-rich motif (residues 309-315). Sequences from *L. erythrorhizon* are red. Nodes in phylograms with support values > 95 are indicated by the grey circles. The full phylogram for the entire FPPS family with branch lengths and leaf labels is available in the Supple- mental. Heatmap showing the gene expression pattern of *L. erythrorhizon* FPPS genes in whole roots (R), arial tissue (A), root periderm (P), root vascular (V), hairy root grown in dark (D), and hairy root grown in the light (L) (b). The cladogram (left) shows evolutionary relationship between FPPS genes according to the overall maximum likelihood phylogeny in part (a).

Fig. 6

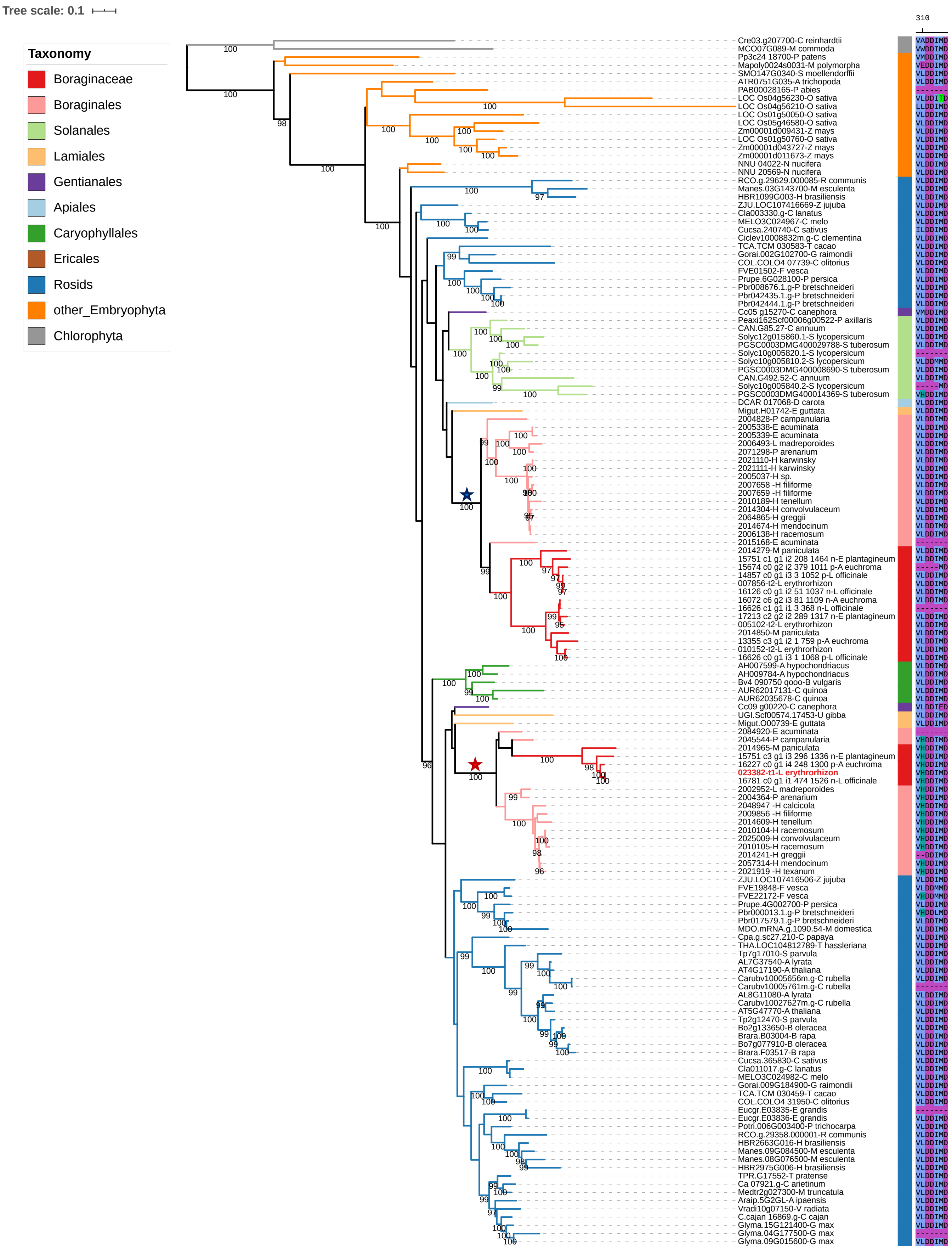

Fig S8 Maximum likelihood phylogeny of FPPS gene family in green plants. Clades representative of subfamily I and II are labeled with a red and blue star, respectively. LeGPPS is shown in red text and an alignment of the Asp-rich motif (residues 309-315) are displayed on the right. Nodes with IQ-TREE support values > 95 are indicated by numbers on the preceding branch. The branches and outer color bar are color-coded to match the taxonomic classification of each sequence. The tree is rooted on Chlorophytes.

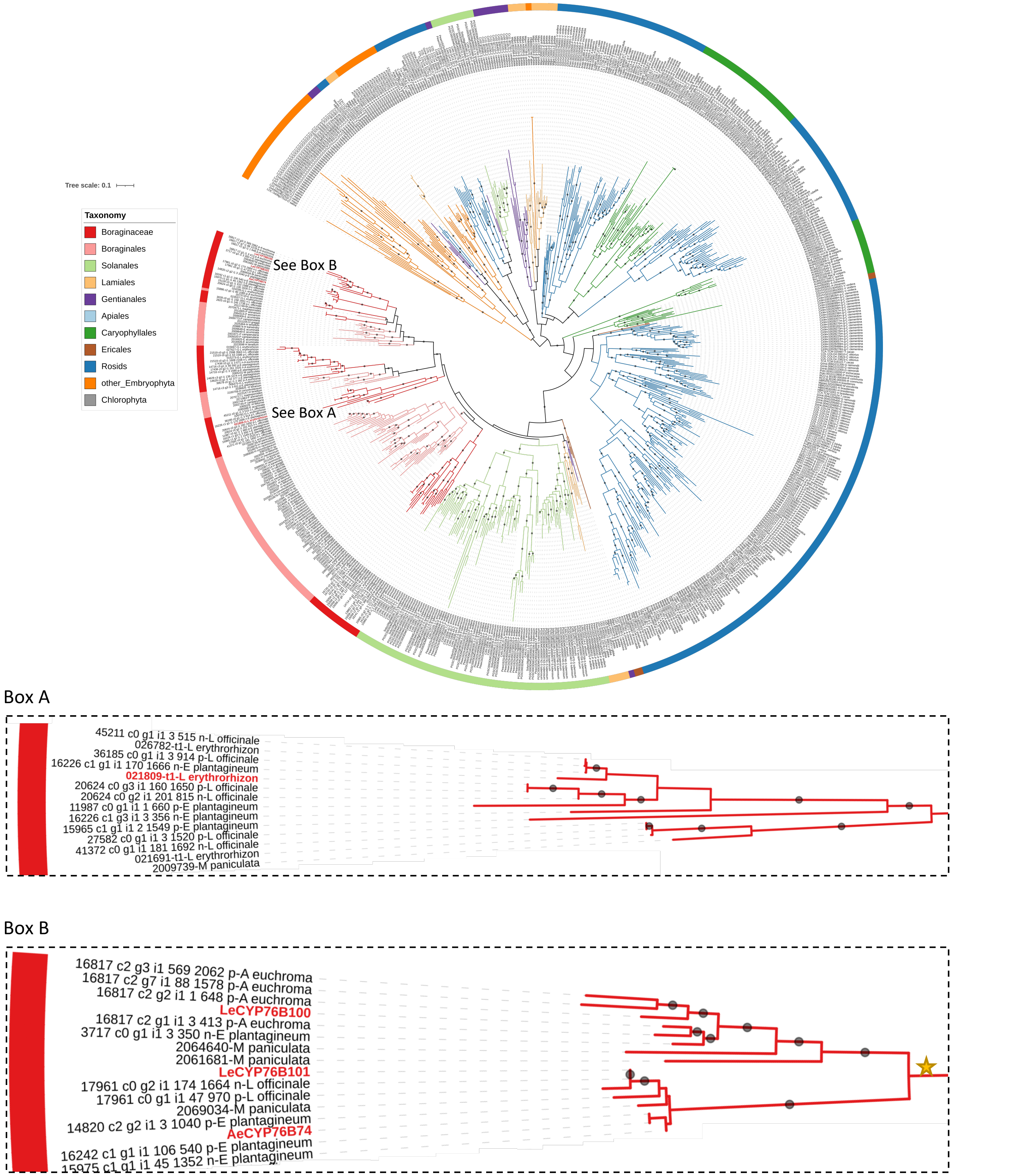

Figure S9 Maximum likelihood phylogeny of cytochrome CYP76B gene family in green plants. Gene candidate Leryth_021809 recovered in the shikonin coexpression network analysis is shown in red text (see box A). Other CYP76B genes, including CYP76B74 in *A. euchroma* and CYP76B100/101 in *L. erythrorhizon* are also shown in red text (see box B). Duplication event giving rise to CYP76B100/101 in *L. erythrorhizon* is indicated with a yellow star. Nodes with IQ-TREE support values > 95 are indicated by grey circles on the preceding branch. The branches and outer color bar are color-coded to match the taxonomic classification of each sequence. The tree is rooted based on rough guide tree of entire cytochrome P450 gene family (Plaza Dicots 4.0 HOM04D000003).

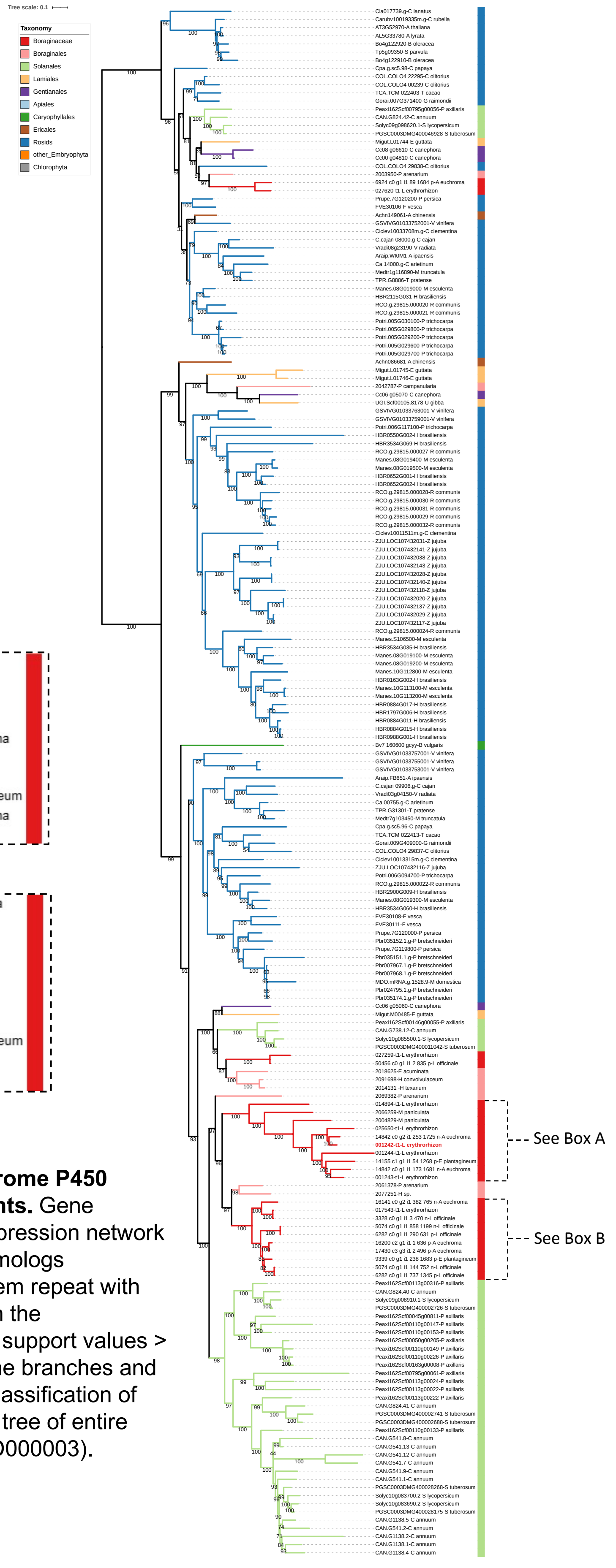

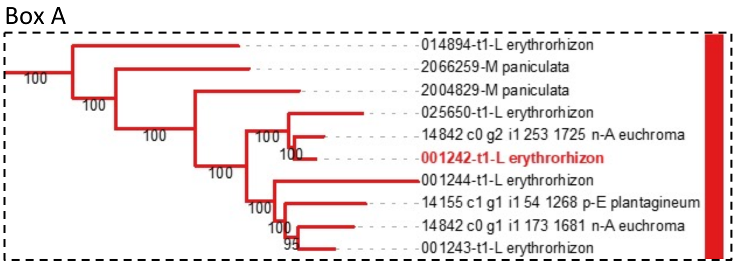

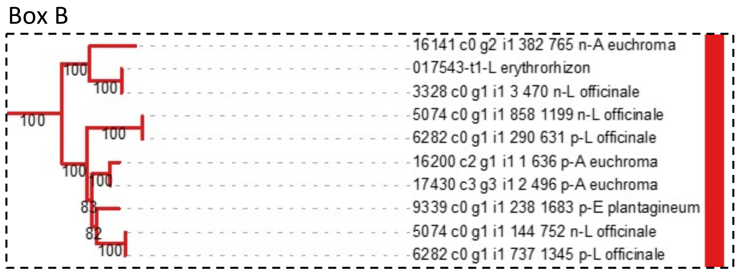

Figure S10 Maximum likelihood phylogeny of cytochrome P450 candidate Leryth_001242 and homologs in green plants. Gene candidate Leryth_001242 recovered in the shikonin coexpression network analysis is shown in red text (see box A). Other P450 homologs Leryth_001243 and Leryth_001244 are present in a tandem repeat with Leryth_001242 (see Fig. S11). An additional expansion in the Boraginaceae is captured in box B. Nodes with IQ-TREE support values > 95 are indicated by numbers on the preceding branch. The branches and outer color bar are color-coded to match the taxonomic classification of each sequence. The tree is rooted based on rough guide tree of entire cytochrome P450 gene family (Plaza Dicots 4.0 HOM04D000003).

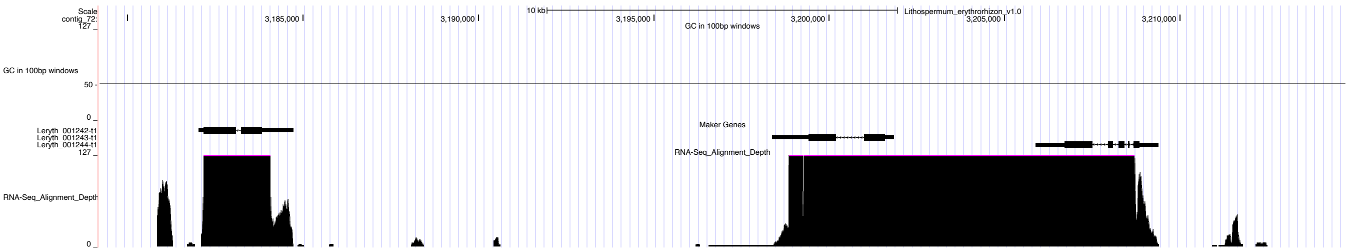

Figure S11 UCSC Genome Browser region for cytochrome P450 candidate Leryth_001242.

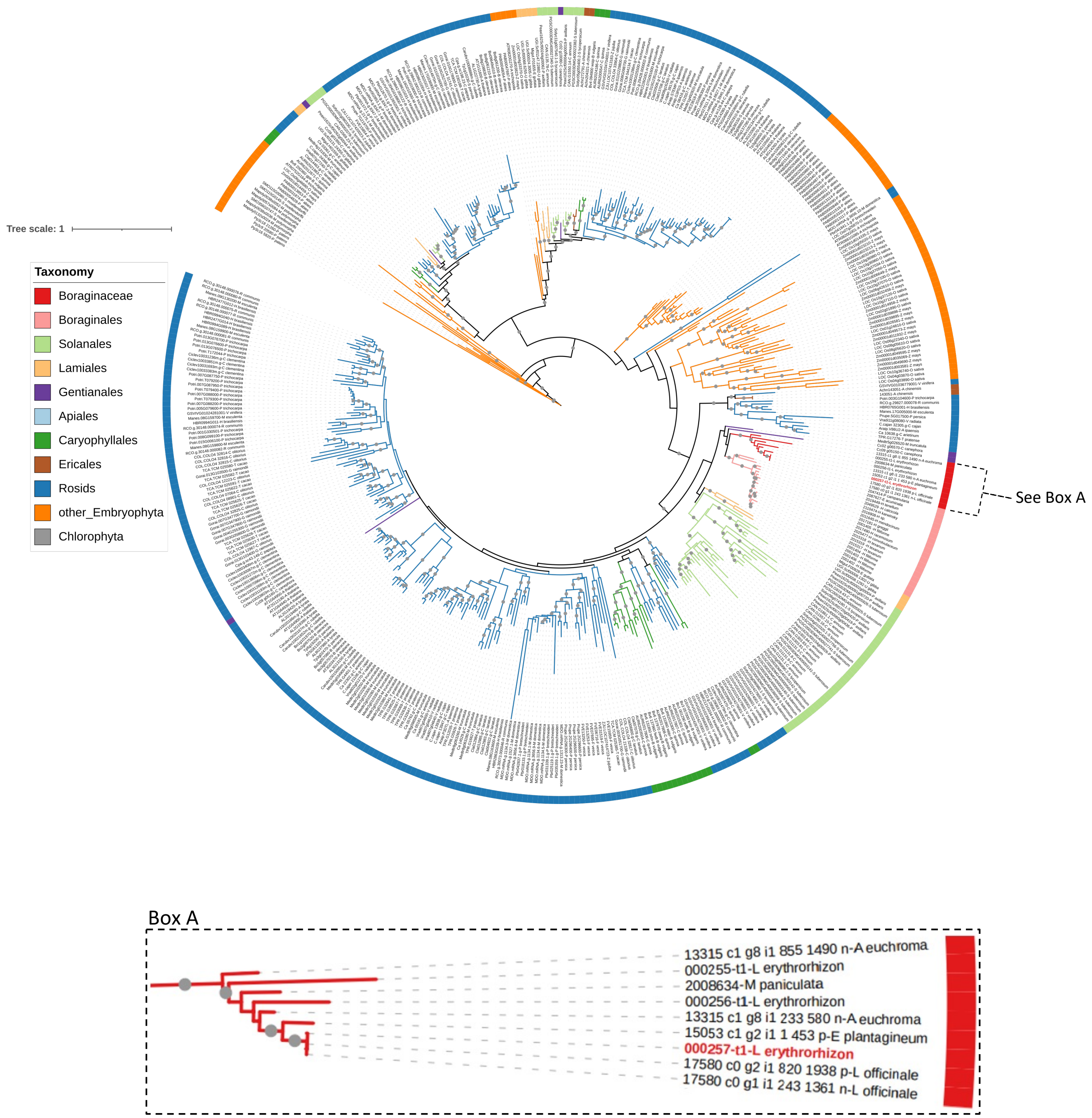

Figure S12 Maximum likelihood phylogeny of cytochrome P450 candidate Leryth_000257 and homologs in green plants. Gene candidate Leryth_000257 recovered in the shikonin coexpression network analysis is shown in red text (see box A). Nodes with IQ-TREE support values > 95 are indicated by grey circles on the preceding branch. The branches and outer color bar are color-coded to match the taxonomic classification of each sequence. Tree is midpoint rooted. The tree is rooted based on rough guide tree of entire cytochrome P450 gene family (Plaza Dicots 4.0 HOM04D000435).

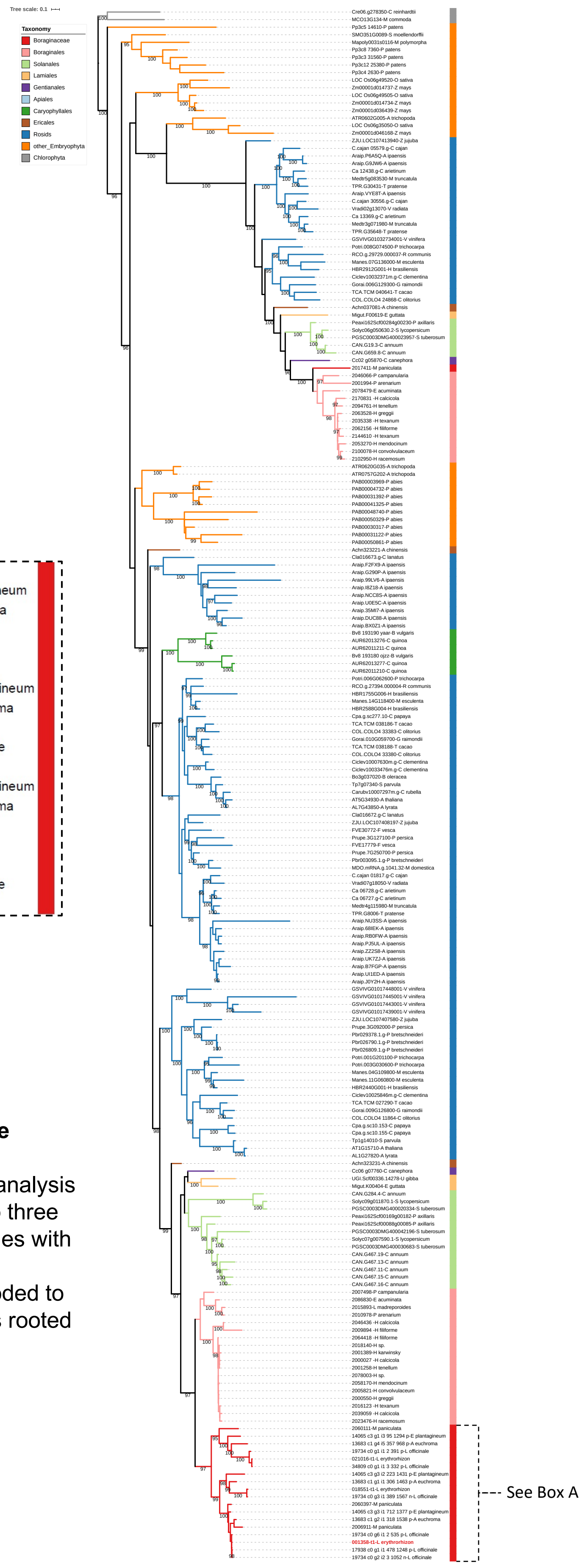

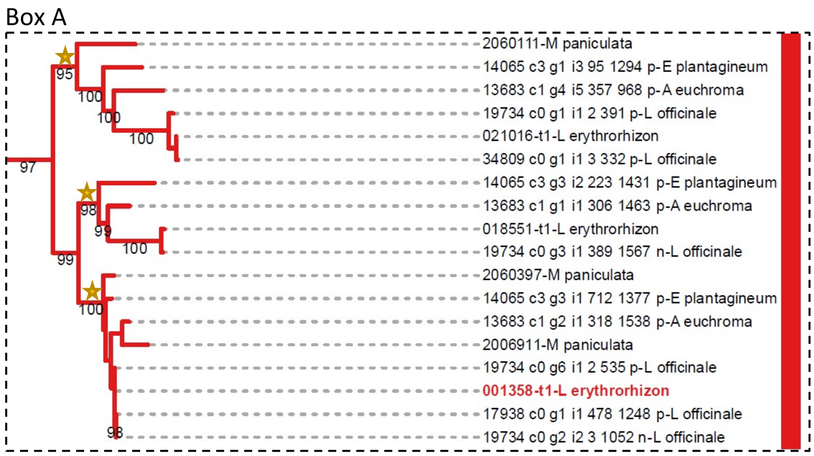

Figure S13 Maximum likelihood phylogeny of the prephenate dehydrogenase gene family in green plants. Gene candidate Leryth_001358 recovered in the shikonin coexpression network analysis is shown in red text (see box A). Duplication events giving rise to three homologs in *L. erythrorhizon* are indicated with yellow stars. Nodes with IQ-TREE support values > 95 are indicated by numbers on the preceding branch. The branches and outer color bar are color-coded to match the taxonomic classification of each sequence. The tree is rooted on Chlorophytes.

Box A

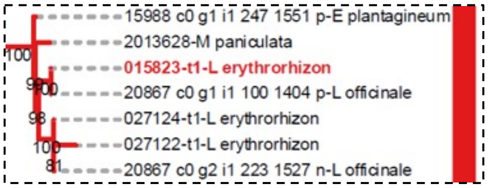

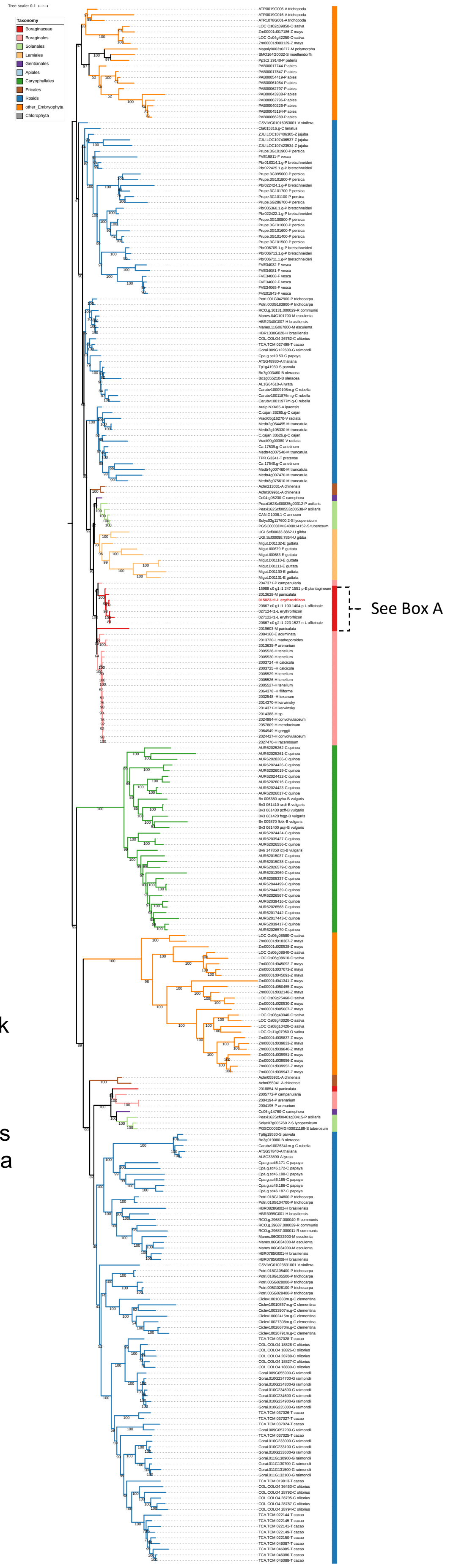

Figure S14 Maximum likelihood phylogeny of transferase candidate Leryth_015823 and homologs in green plants. Gene candidate Leryth_0015823 recovered in the shikonin coexpression network analysis is shown in red text (see box A). Nodes with IQ-TREE support values > 95 are indicated by numbers on the preceding branch. The branches and outer color bar are color-coded to match the taxonomic classification of each sequence. The tree is rooted based on rough guide tree of entire AT gene family (Plaza Dicots 4.0 HOM04D000075).

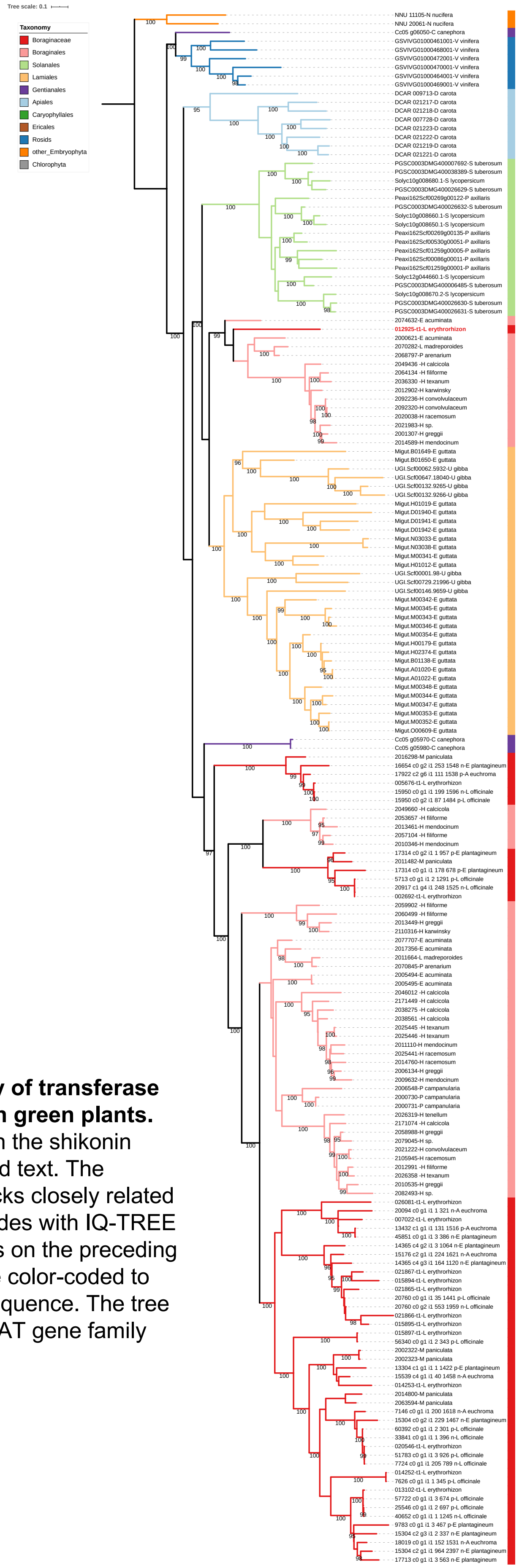

Figure S15 Maximum likelihood phylogeny of transferase Candidate Leryth_012925 and homologs in green plants. Gene candidate Leryth_0012925 recovered in the shikonin coexpression network analysis is shown in red text. The candidate is present on a long branch and lacks closely related homologs in other Boraginaceae species. Nodes with IQ-TREE support values > 95 are indicated by numbers on the preceding branch. The branches and outer color bar are color-coded to match the taxonomic classification of each sequence. The tree is rooted based on rough guide tree of entire AT gene family (Plaza Dicots 4.0 HOM04D000339).

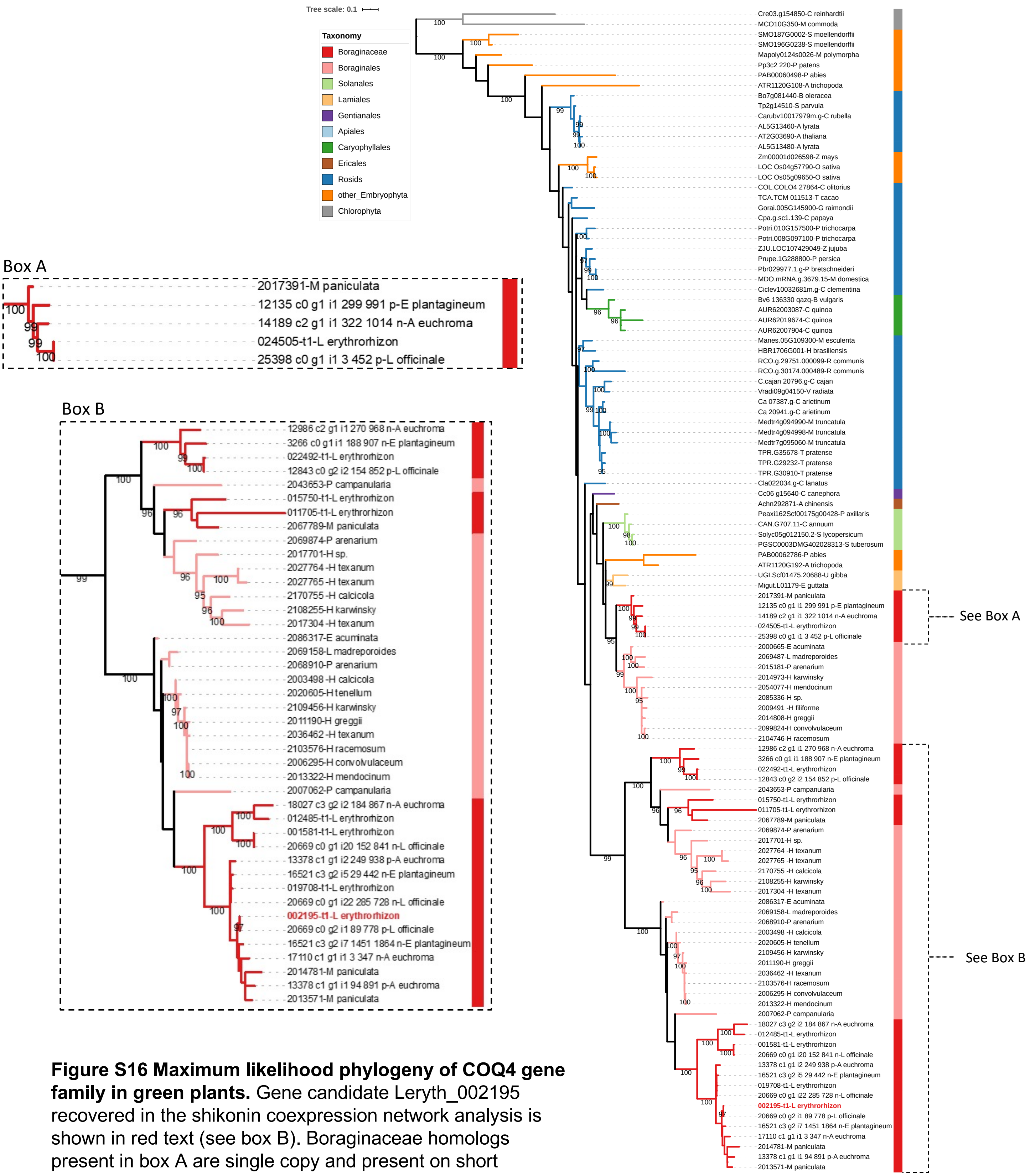

Figure S16 Maximum likelihood phylogeny of COQ4 gene family in green plants. Gene candidate Leryth_002195 recovered in the shikonin coexpression network analysis is shown in red text (see box B). Boraginaceae homologs present in box A are single copy and present on short branches. Box B indicates a Boraginales-specific gene family expansion of COQ4 homologs, with longer branches compared to those in box A. Nodes with IQ-TREE support values > 95 are indicated by numbers on the preceding branch. The branches and outer color bar are color-coded to match the taxonomic classification of each sequence. The tree is rooted on Chlorophytes.

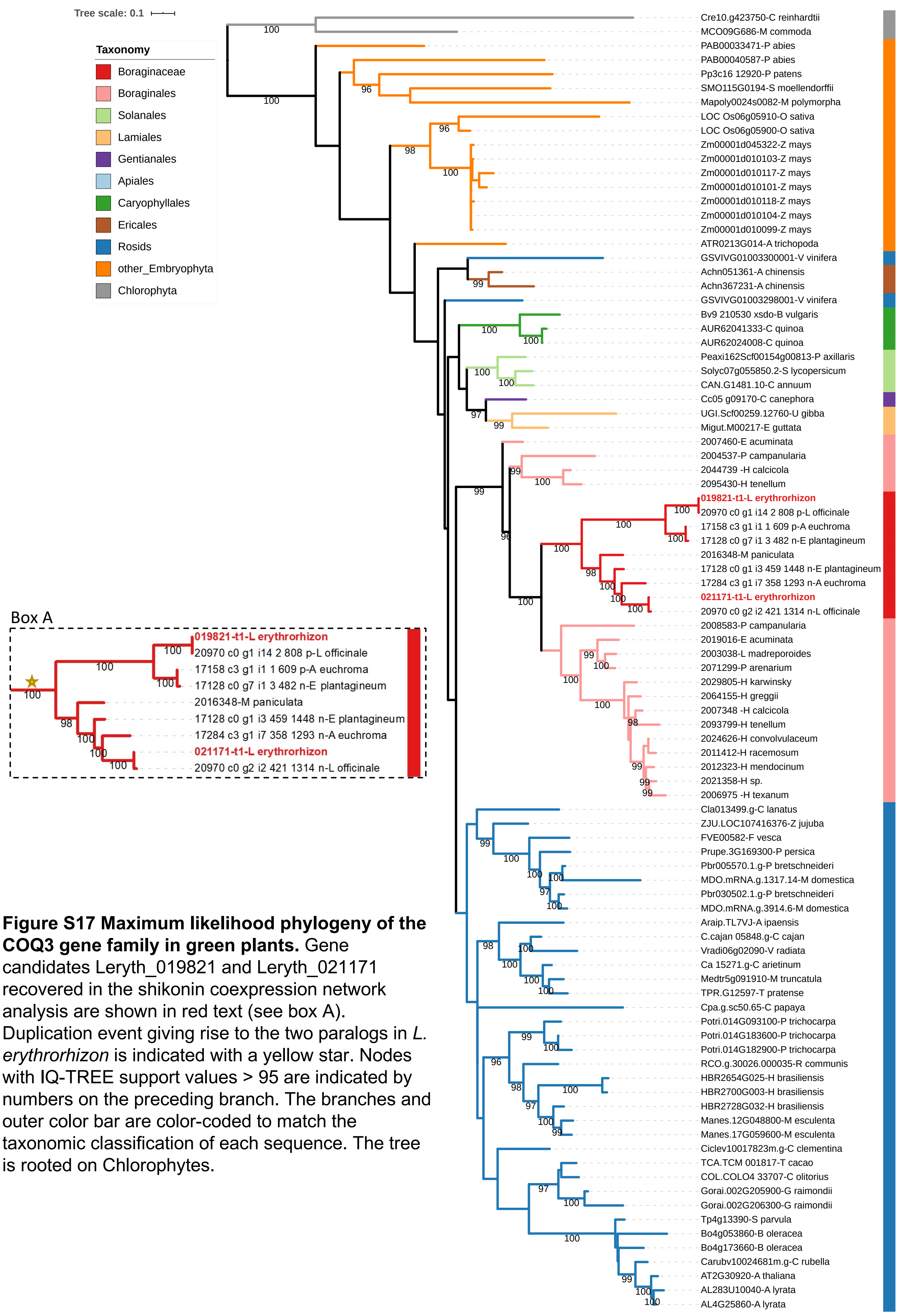

Figure S17 Maximum likelihood phylogeny of the COQ3 gene family in green plants. Gene candidates Leryth_019821 and Leryth_021171 recovered in the shikonin coexpression network analysis are shown in red text (see box A). Duplication event giving rise to the two paralogs in *L. erythrorhizon* is indicated with a yellow star. Nodes with IQ-TREE support values > 95 are indicated by numbers on the preceding branch. The branches and outer color bar are color-coded to match the taxonomic classification of each sequence. The tree is rooted on Chlorophytes.

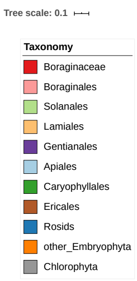

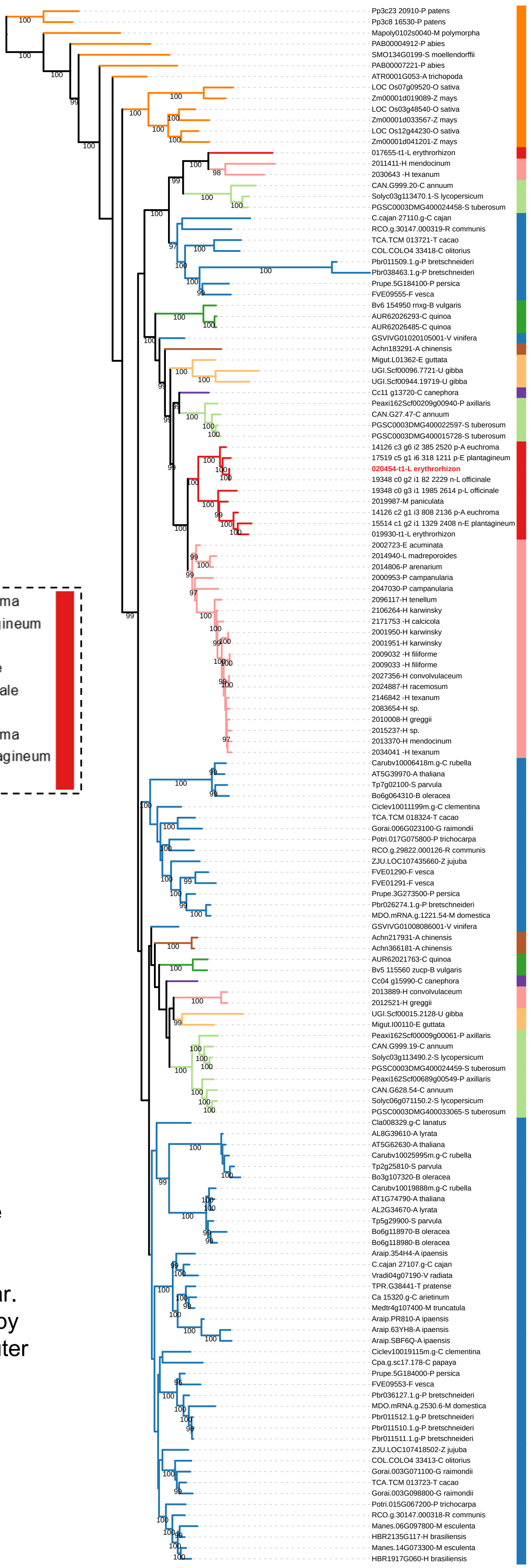

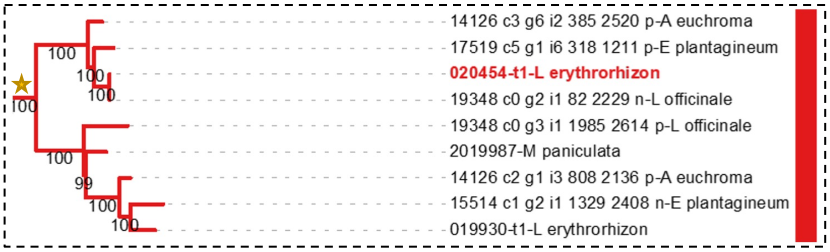

Figure S18 Maximum likelihood phylogeny of quinoprotein dehydrogenase gene family in green plants. Gene candidate Leryth_020454 recovered in the shikonin coexpression network analysis is shown in red text (see box A). Duplication event giving rise to two paralogs in *L. erythrorhizon* is indicated with a yellow star. Nodes with IQ-TREE support values > 95 are indicated by numbers on the preceding branch. The branches and outer color bar are color-coded to match the taxonomic classification of each sequence. The tree is rooted on mosses.

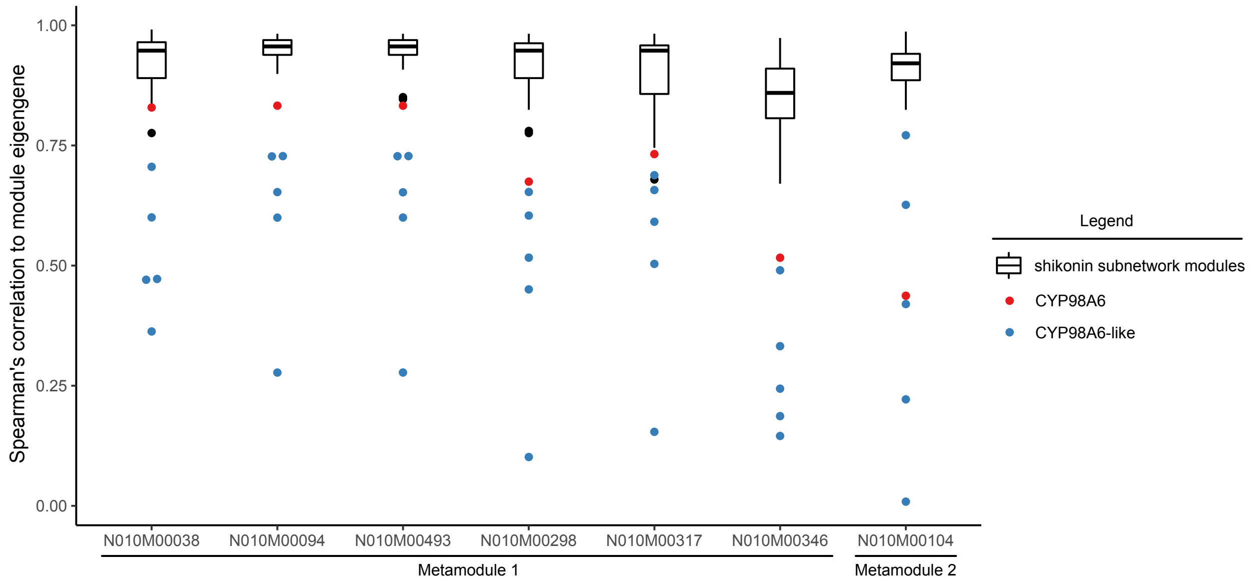

Figure S19 Spearman's correlation to module eigengenes of shikonin subnetwork. The box and whisker plots show the distribution of spearman correlation of coexpression (SCC) of each module eigengene against every gene within the module. The red points show the SCC of each eigengene against the *CYP98A6* (Leryth_006600) rosmarinic acid biosynthesis gene. The blue points show the SCC of each eigengene against five additional *CYP98A6*-like genes. The correlation of *CYP98A6* and *CYP98A6*-like genes are outside the third interquartile range for each module.

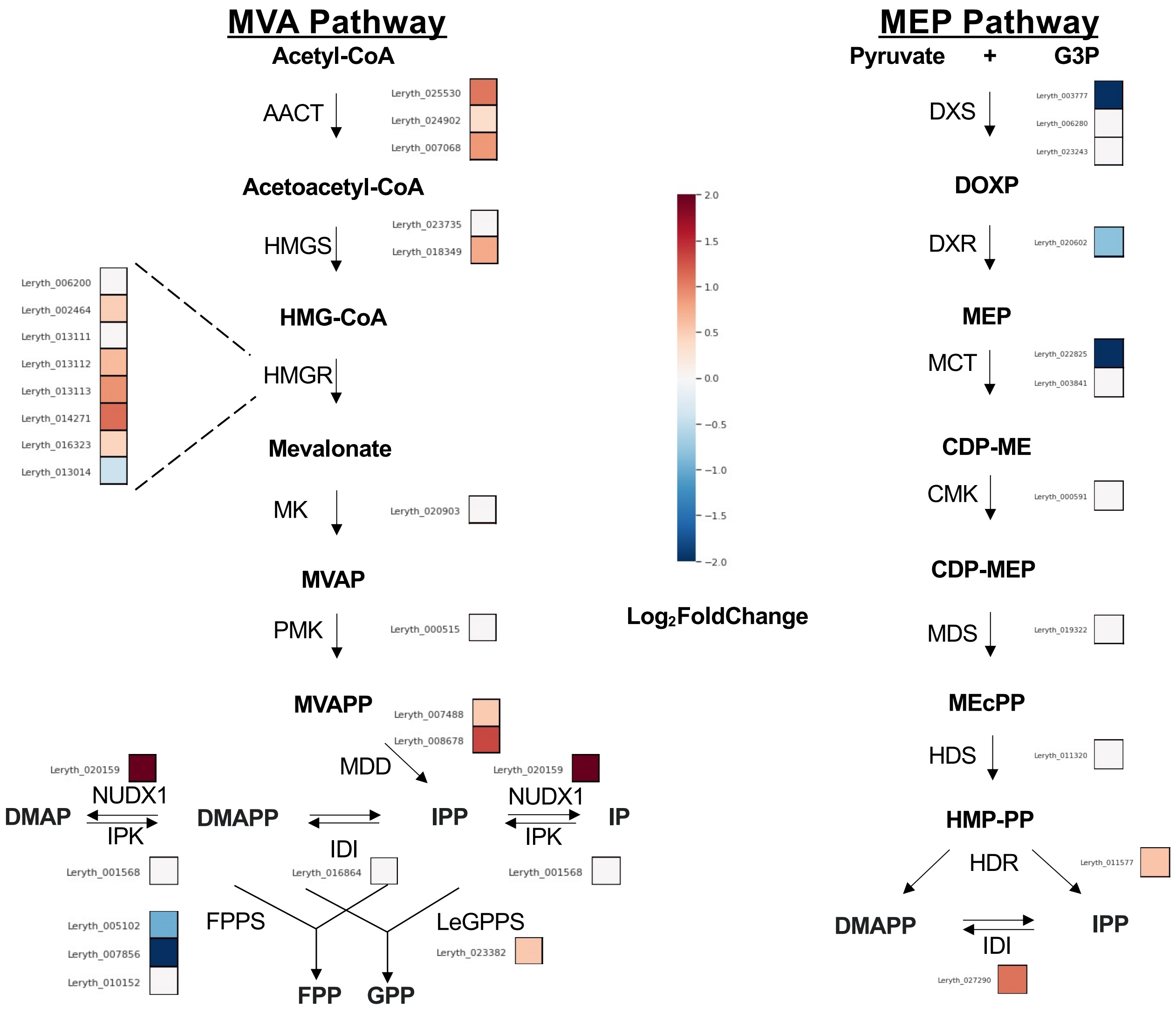

Figure S20 Effect of mevinolin treatment on expression of MVA and MEP pathway genes in *L. erythrorhizon* hairy roots. The average $\log_2$fold-change in expression for each gene in mevinolin treated versus control *EV-26* lines in the mevalonic acid (MVA) and methylerythritol phosphate (MEP) pathways. Mock (control) and 100 µM mevinolin treatments were administered immediately upon transfer of 14-d-old hairy roots to M9 and darkness. Total RNA was extracted at 6 d after transfer of 14-d-old hairy roots to M9 and darkness and used for RNA-seq analysis. See Fig. 4 legend for abbreviations.

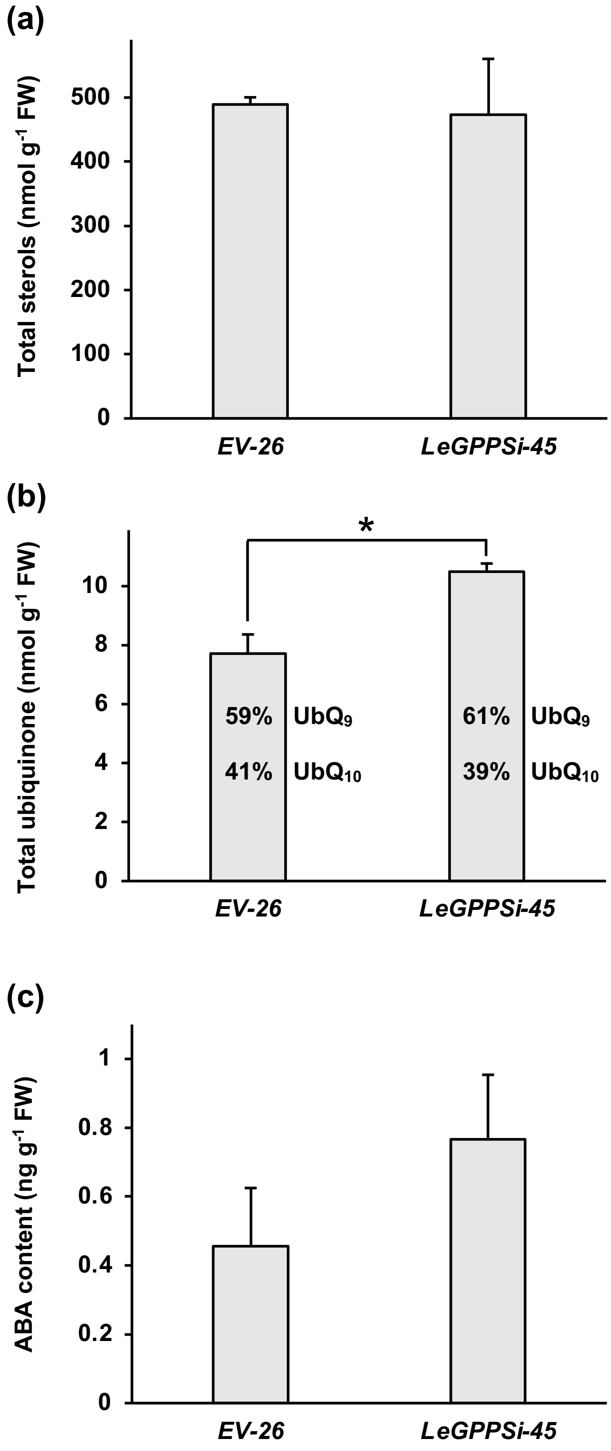

Figure S21 Pool sizes of sterols (a), ubiquinones (b), and abscisic acid (ABA) (c) measured in empty-vector control line 26 (*EV*-26) and *LeGPPS* RNAi line 45 (*LeGPPSi*-45). Metabolites were measure at 6 d after transfer of 14-d-old hairy roots to M9 and darkness. All data are means ± SEM (n = 3–4 biological replicates). Statistically significant differences are indicated (*P < 0.05, Student's *t* test).
